# Assessment of the Rapid Shallow Breathing Index, Integrative Weaning Index, and Dead Space to Tidal Volume Ratio by Respiratory Failure Type in Successfully Weaned Emergency Department Patients

**DOI:** 10.3390/medicina61081438

**Published:** 2025-08-10

**Authors:** Murtaza Kaya, Harun Yildirim, Ali Halici, Abdil Coskun, Mehmed Ulu, Mehmet Toprak, Sami Eksert

**Affiliations:** 1 Department of Emergency Medicine, Medical Faculty, Kutahya Health Sciences University, 43000 Kutahya, Turkey; harun.yildirim@ksbu.edu.tr (H.Y.); ali.halici@ksbu.edu.tr (A.H.); abdil.coskun@ksbu.edu.tr (A.C.); 2 Department of Emergency Medicine, Adiyaman Training and Research Hospital, 02030 Adiyaman, Turkey; mehmed.ulu@ksbu.edu.tr; 3 Department of Emergency Medicine, Kutahya City Hospital, 43040 Kutahya, Turkey; mehmet.toprak@ksbu.edu.tr; 4 Department of Anesthesiology and Reanimation, Gulhane Training and Research Hospital, Health Sciences University, 06010 Ankara, Turkey; sami.eksert@sbu.edu.tr

**Keywords:** weaning, mechanical ventilator, respiratory dead space, tidal volume, respiratory failure

## Abstract

*Background/Objectives*: Mechanical ventilation is essential in the management of acute respiratory failure (RF); however, prolonged use increases the risk of complications. Accurate predictors are therefore needed to guide timely weaning. The Rapid Shallow Breathing Index (RSBI), the dead space to tidal volume ratio (VD/VT), and the Integrative Weaning Index (IWI) are among the key indices used to assess weaning readiness. This study aimed to examine whether these indices differ between patients with Type 1 (hypoxemic) and Type 2 (hypercapnic) respiratory failure who were successfully extubated in the emergency department, in order to explore their physiologic variability across respiratory failure phenotypes. *Methods*: This cross-sectional study included 35 adult patients (23 with Type 1 RF, 12 with Type 2 RF) who were successfully weaned from mechanical ventilation in the Emergency Department of a tertiary care hospital between 2022 and 2024. RSBI, VD/VT, IWI, and arterial blood gas parameters were recorded. Descriptive and comparative statistical analyses were performed, with significance set at *p* < 0.05. *Results*: There were no significant differences in age, gender, or comorbidities between the groups. Type 2 RF patients had higher FiO_2_ requirements (37.5% vs. 30.0%; *p* = 0.03) and PaCO_2_ levels (49.1 ± 9.65 mmHg vs. 40.3 ± 4.49 mmHg; *p* < 0.001). The PaO_2_/FiO_2_ ratio was lower in the Type 2 group (169 ± 49.6) compared to the Type 1 group (244 ± 95.6; *p* = 0.017). VD/VT ratios were significantly higher in Type 2 RF patients (0.37 ± 0.04 vs. 0.29 ± 0.13; *p* = 0.046). RSBI values were identical between groups (40.0 in both; *p* = 1.00), and IWI values showed no significant difference (70.8 ± 30.7 vs. 79.3 ± 32.5; *p* = 0.45). *Conclusions*: Although RSBI and IWI values were similar across respiratory failure types, patients with Type 2 RF demonstrated higher VD/VT ratios and lower PaO_2_/FiO_2_, indicating reduced gas exchange and alveolar ventilation efficiency. These findings suggest that VD/VT may be a more useful parameter for assessing weaning readiness in hypercapnic patients.

## 1. Introduction

Mechanical ventilation (MV) is one of the essential interventions commonly used on patients with Acute Respiratory Failure (RF) to keep them alive [1]. However, MV may cause the development of ventilator-associated pneumonia, muscle mass depletion, and increased mortality [2]. Consequently, timely and successful separation from MV support reduces the side effects and increases the chances of patients’ survival. Weaning is the process when the patient is switched from MV to spontaneous breathing. This complex procedure needs close monitoring and accurate judgments of the patient’s condition to ensure effective extubation [3].

Weaning from the MV uses several indices that predict the patient’s chances of passing through the process. Among these output indices, the most widely used index is the Rapid Shallow Breathing Index (RSBI) [4]. Respiratory rate to Tidal Volume (TV) in patients is measured during spontaneous breathing trials and is termed as RSBI [5]. However, other than RSBI, there is a new valuable index known as VD/VT, utilized in evaluating the efficiency of the lungs in terms of gas exchange in RF patients. This ratio is regarded as the less frequent indicator of V/P inequality and is particularly important for hypercapnic RF. It may be used to determine the extent of the patient’s capacity for adequate oxygenation or CO_2_ removal without invasive MV [6]. Additionally, the Integrative Weaning Index (IWI), which combines static compliance, oxygenation, and the RSBI into a single composite measure, has been proposed to enhance prediction accuracy. However, despite its theoretical advantages, studies such as the one by Boniatti et al. have shown that modified IWI does not reliably predict extubation failure, limiting its clinical utility in critical care settings. This highlights the need for further investigation into its applicability across different types of respiratory failure [7].

Although these indices are common, they can fail to predict a weaning success accurately. It has to be noted that traditional identifiers such as RSBI might not capture alterations in respiratory mechanics or pathophysiologic variation among various RF kinds [8]. RF is classified into two categories: Type 1, Hypoxemic RF, and Type 2, Hypercapnic RF. Type 1 RF, which is associated with an impaired oxygen exchange, can occur in Acute Respiratory Distress Syndrome (ARDS), Pneumonia, and Pulmonary Embolism (PE) [9]. Type 2 RF results from alveolar hypoventilation and may develop in conditions such as Chronic Obstructive Pulmonary Disease (COPD), Neuromuscular Disorders, and Obesity Hypoventilation Syndrome (OHS). The severity of these two types of respiratory failure differs in their pathophysiology and responsiveness to ventilatory support, which may affect the weaning success [10].

The past literature reviews on MV weaning strategies have highlighted some areas where there is limited understanding of differentiating the type of RF based on weaning indices. There is insufficient literature on the sensitivity and specificity of RSBI and VD/VT alone or in identifying patients with different RF subtypes, typically in patients successfully weaned from MV [11,12]. Research in this area has mainly focused on general patient sampling without differentiating the pathophysiology of Type 1 and Type 2 RF. This has confused the best weaning indicators for each type of RF and how to correctly use these markers to aid in clinical decision-making, especially in the Emergency Department (ED). In addition, the interactions between RSBI, VD/VT, other weaning parameters, and extubation success have not been well clarified in ED-based populations.

The present study aimed to investigate whether commonly used weaning indices—RSBI, VD/VT, and IWI—differ according to the type of respiratory failure (Type 1 vs. Type 2) in patients who were successfully extubated in the emergency department. By examining these indices in relation to patient characteristics and gas exchange parameters, this study seeks to clarify their applicability across different respiratory failure phenotypes.

## 2. Materials and Methods

### 2.1. Study Design and Setting

This cross-sectional study was conducted between 2022 and 2024 in the Emergency Department of a tertiary care hospital with an annual patient volume of approximately 250,000 visits. Our emergency department encompasses a dedicated critical care area, ensuring thorough evaluation and continuous monitoring of patients requiring mechanical ventilation.

### 2.2. Ethical Consideration

The study was approved by the Non-Interventional Clinical Research Ethics Committee of Kutahya Health Sciences University (Approval No: 2021/08-11, Date: 28 April 2021). Written informed consent was obtained from all participants or their legal representatives prior to enrollment. Patient confidentiality was maintained throughout the study.

### 2.3. Participants and Recruitment

**Figure 1:**
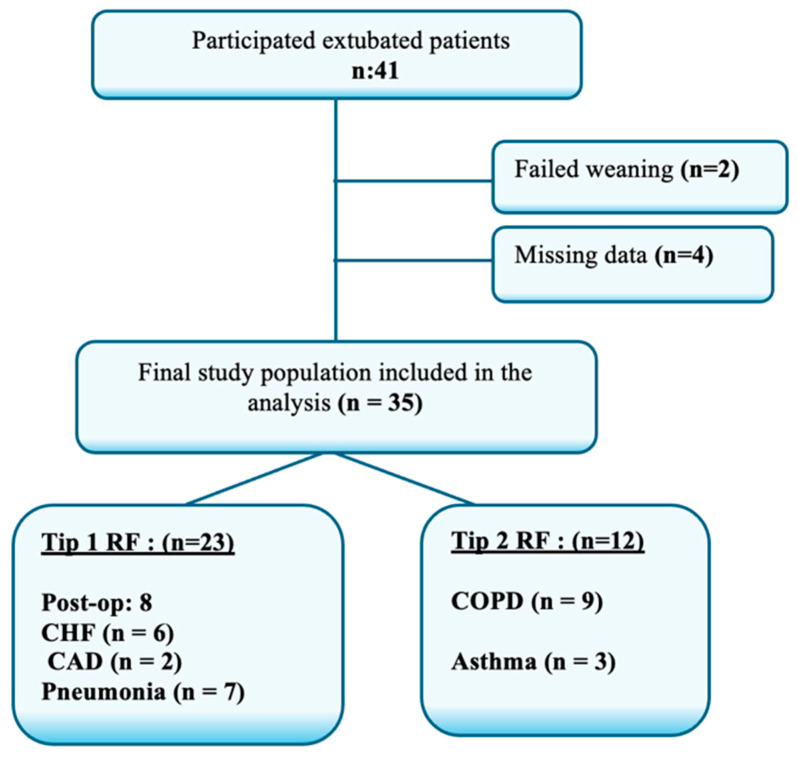
Flow Chart of Study Population. RF: Respiratory Failure, Post-op: Postoperative Patients, CHF: Congestive Heart Failure, CAD: Coronary Artery Disease, COPD: Chronic Obstructive Pulmonary Disease.

The study included adult patients (≥18 years) who were successfully weaned from mechanical ventilation. Successful weaning was defined as maintaining spontaneous breathing without the need for reintubation or non-invasive ventilation for at least 48 h following extubation. A total of 35 patients met the inclusion criteria and were categorized into two groups based on the type of respiratory failure (RF): 23 patients with Type 1 RF (hypoxemic) and 12 patients with Type 2 RF (hypercapnic). Among the Type 1 RF group, 8 patients were postoperative surgical cases, 6 had congestive heart failure, 2 had coronary artery disease, and 7 had pneumonia. In the Type 2 RF group, 9 patients were diagnosed with chronic obstructive pulmonary disease (COPD) and 3 with asthma. Patients with missing data or who required any form of mechanical ventilatory support within 48 h post-extubation were excluded. A flow diagram summarizing the eligibility and inclusion process is presented in Figure 1. All extubated patients were discharged following ward follow-up, and all exhibited stable mental status.

### 2.4. Data Collection

The following variables were recorded at the time of admission: age, gender, heart rate, respiratory rate, systolic blood pressure (SBP), hemoglobin (Hb), blood urea nitrogen (BUN), and serum creatinine. During the period of mechanical ventilation, respiratory mechanics and ventilatory settings were monitored, including positive end-expiratory pressure (PEEP), peak inspiratory pressure (Pmax), plateau pressure (Pplato), tidal volume (TV), and both static and dynamic lung compliance. Arterial blood gas (ABG) analysis was used to assess pH, partial arterial oxygen pressure (PaO_2_), and partial arterial carbon dioxide pressure (PaCO_2_).

### 2.5. Weaning Procedure

Following initial endotracheal intubation, patients were managed in volume-controlled assist-control mode (V-ACV). Once sedation was reduced and spontaneous respiratory efforts were observed, ventilator mode was transitioned to volume-synchronized intermittent mandatory ventilation with pressure support (V-SIMV + PS). During this phase, we closely monitored the tidal volume of spontaneous breaths. If spontaneous tidal volumes exceeded 250 mL, the backup respiratory rate was gradually reduced to 10 breaths per minute to allow spontaneous ventilation to dominate. As spontaneous tidal volumes improved, patients were switched to pressure support ventilation (PSV). When patients became more alert and demonstrated sufficient ventilatory effort, extubation was performed, typically after the administration of corticosteroids and antiemetics to reduce post-extubation complications such as stridor or vomiting. Weaning indices were assessed immediately before extubation and included the following:

- RSBI: Derived from respiratory rate and tidal volume and presented as a fractional value of f/VT.
- Dead Space to Tidal Volume Ratio (VD/VT): Measured as (PaCO_2_ − End TidalCO_2_)/PaCO_2_.
- Integrative Weaning Index (IWI): Static compliance × Oxygen saturation SaO_2_/RSBI.

### 2.6. Statistical Analysis

All statistical analyses described in this manuscript were conducted using the SPSS statistical tools, version 27 software (IBM, Armonk, NY, USA). Data for continuous variables were presented as mean ± Standard Deviation (SD) or median Interquartile Range (IQR) based on the distribution. Categorical variables were expressed as frequencies and percentages. Comparisons between groups were made using Student’s *t*-test for independent samples for continuous variables with a normal distribution and the Mann–Whitney U test for non-continuous variables with a skewed distribution. Categorical data were analyzed using the Chi-square or Fischer exact test, whichever was applied. In this study, statistical significance was determined at *p*-value < 0.05.

## 3. Results

A total of 35 patients successfully weaned from mechanical ventilation were included in the study. Of these, 23 patients were classified as having Type 1 RF, and 12 had Type 2 RF T. The demographic and baseline clinical characteristics of the two groups are summarized in Table 1.

The mean age of patients in both groups was similar, for Type 1 RF = 72.57 ± 18:2 years for Type 2 RF = 71.75 ± 8.7 years, respectively, *p* = 0.88. Males had higher representation in both groups, 69.6% for type 1 RF and 83.3% for type 2 RF, with *p* = 0.37. Systolic Blood Pressure (SBP) did not differ significantly between the two kinds of RF. Type 1 RF had a higher heart rate of 97.52 ± 14.19 bpm than Type 2 RF, with a heart rate of 88.25 ± 14.34 bpm, even though the difference was not statistically significant, with *p* = 0.07. SBP was increased in Type 2 RF patients, 130.17 ± 21.95 mmHg, compared to Type 1 RF patients, 122.96 ± 14.59 mmHg, but that was not statistically significant (*p* = 0.25).

The respiration rate, blood hemoglobin, Blood Urea Nitrogen (BUN), and creatinine did not demonstrate any intergroup differences. These Median BUN values were 28.00 mg/dL for Type 1 RF and 37.00 mg/dL for Type 2 RF with *p* = 0.70. The values of median creatinine were 1.16 mg/dL and 0.97 mg/dL, respectively, with *p* = 0.56, as shown in Table 1.

The results of MV parameters and ABG analysis are illustrated in Table 2. The FiO_2_ requirement was significantly higher in the Type 2 RF group with median = 37.50% and IQR = 25.5–40 compared to the Type 1 RF group with median = 30.0% and IQR = 25.5–35 (*p* = 0.03). Other ventilatory parameters, including PEEP, Pmax, Pplato, VT, and compliance (static and dynamic), were similar between the groups. ABG analysis revealed a significantly higher PaCO_2_ level in the Type 2 RF group (mean: 49.1 ± 9.65 mmHg) compared to the Type 1 RF group (mean: 40.3 ± 4.49 mmHg) (*p* < 0.001), consistent with the hypercapnic nature of Type 2 RF.

Weaning indices, including RSBI, VD/VT, and PaO_2_/FiO_2_, are presented in Table 3. The RSBI values were identical in both groups, with median = 40.0 and IQR: 35–40 (*p* = 1.00). However, the VD/VT ratio was significantly higher in the Type 2 RF group (mean 0.37 ± 0.04) compared to the Type 1 RF group with mean 0.29 ± 0.13 (*p* = 0.046). The PaO_2_/FiO_2_ ratio was significantly lower in the Type 2 RF group with a mean of 169 ± 49.6 mmHg compared to the Type 1 RF group with a mean of 244 ± 95.6 mmHg (*p* = 0.017). Other indices, including the IWI, PaO_2_/PAO_2_ ratio, and GCS scores after extubation showed no statistically significant differences between the groups.

## 4. Discussion

The unique contribution of this study lies in the separate evaluation of weaning indices based on the type of respiratory failure—Type 1 (hypoxemic) and Type 2 (hypercapnic)—in patients who were successfully weaned from mechanical ventilation. While most studies examine these parameters in heterogeneous ICU populations, our analysis focused on how pathophysiological differences between respiratory failure phenotypes may influence the predictive value of indices such as RSBI, VD/VT, and IWI. Mechanical ventilation (MV) is essential for managing acute respiratory failure (RF), but prolonged use increases the risk of complications [13]. Therefore, timely and accurate prediction of weaning readiness is critical for patient outcomes. In our study, the VD/VT ratio was significantly higher in patients with Type 2 RF, suggesting impaired alveolar ventilation and increased physiological dead space—a finding consistent with the gas exchange inefficiency commonly observed in hypercapnic conditions such as obstructive pulmonary diseases [14,15,16,17].

Despite its widespread clinical use, the predictive value of the Rapid Shallow Breathing Index (RSBI) remains controversial, particularly when applied as a stand-alone test. In a comprehensive meta-analysis by Trivedi et al. involving over 10,000 critically ill patients, RSBI demonstrated moderate sensitivity (0.83) but poor specificity (0.58) for predicting successful extubation. These findings remained consistent across different RSBI thresholds (<80, 80–105, >105), highlighting its limited discriminatory capacity [18]. Such limitations emphasize the need for more physiologically integrated parameters in assessing extubation readiness.

Supporting this, a recent multicenter study by Vahedian-Azimi et al. compared the diagnostic accuracy of RSBI, IWI, and several other weaning indices. Among these, IWI demonstrated the highest sensitivity and specificity, clearly outperforming RSBI in predicting weaning success [19]. The authors concluded that IWI offers a more comprehensive assessment of respiratory mechanics, gas exchange, and patient effort, making it a more reliable tool in clinical decision-making regarding ventilator liberation.

In the present study, the VD/VT ratio was significantly higher in patients with Type 2 RF compared to those with Type 1 RF (0.37 ± 0.04 vs. 0.29 ± 0.13; *p* = 0.046). This finding aligns with the observation by Lazzari et al., who reported that increased physiological dead space is associated with reduced ventilation efficiency [20]. In hypercapnic individuals, impaired alveolar ventilation due to ventilation-perfusion mismatch contributes to elevated VD/VT ratios. Similarly, Jiang et al. found that VD/VT ratios above 0.6 were associated with increased mortality, emphasizing the clinical relevance of dead space measurements [21].

Furthermore, the PaO_2_/FiO_2_ ratio also differed significantly between groups, with values of 244 ± 95.6 in the Type 1 RF group and 169 ± 49.6 in the Type 2 RF group (*p* = 0.017). This result is consistent with Abbott et al., who highlighted that lower PaO_2_/FiO_2_ ratios are indicative of more severe gas exchange impairment in hypoxemic respiratory failure [22]. Chiumello et al. further supported this by reporting that a ratio below 200 is associated with poor clinical outcomes [23]. The lower PaO_2_/FiO_2_ ratio in the Type 2 RF group in our study thus underscores the severity of gas exchange dysfunction in hypercapnic patients.

The mean RSBI in our study was 40.0 and did not differ significantly between the groups (*p* = 1.00), indicating its limited prognostic value in hypercapnic patients. Supporting this, Ghiani et al. reported that RSBI had poor predictive accuracy for spontaneous breathing trial failure (AUROC: 0.54) in prolonged, tracheotomized patients. Although IWI outperformed RSBI in their cohort, it still demonstrated only modest discriminatory power (AUROC: 0.66), suggesting the need for more comprehensive and robust indicators of weaning readiness [24].

Sterr et al. emphasized that indices such as RSBI, PaO_2_/FiO_2_, and diaphragm ultrasonography have been widely studied in the context of weaning failure [25]. They highlighted the central role of gas exchange impairment and altered respiratory mechanics, particularly in patients with Type 2 respiratory failure. In our cohort, RSBI values were identical across groups (40.0 ± 14.9 in Type 1 RF vs. 40.0 ± 14.7 in Type 2 RF; *p* = 1.000), underscoring its limited discriminatory capacity. In contrast, PaO_2_/FiO_2_ ratios were significantly lower in the Type 2 RF group (169 ± 49.6 vs. 244 ± 95.6; *p* = 0.017), consistent with the notion of more severe gas exchange impairment in hypercapnic patients. These findings align with Kamal et al., who noted that RSBI and PaO_2_/FiO_2_, when used alone, provide limited predictive power and suggested integrating them with additional physiological markers to improve weaning assessment [26].

The significantly higher VD/VT ratio observed in patients with Type 2 respiratory failure reflects impaired gas exchange efficiency, primarily due to increased physiological dead space. This finding is consistent with the observations of Akella et al., who emphasized the roles of increased airway resistance, intrinsic PEEP, and diaphragmatic dysfunction in weaning failure [27]. In our study, patients with Type 2 respiratory failure exhibited significantly higher PaCO_2_ levels (49.1 ± 9.65 mmHg vs. 40.3 ± 4.49 mmHg; *p* < 0.001) and required higher inspired oxygen concentrations (FiO_2_: 37.5% vs. 30.0%; *p* = 0.03) compared to those with Type 1 respiratory failure. These findings indicate compromised ventilatory efficiency and more severe gas exchange impairment in the hypercapnic group. Despite the absence of significant differences in static and dynamic compliance values between the groups, the elevated PaCO_2_ suggests the presence of alveolar hypoventilation or ventilation-perfusion mismatch, despite similar mechanical settings. This observation aligns with the results of Depta et al., who demonstrated that reductions in VD/VT during PEEP titration were associated with improved gas exchange in ARDS patients [28]. Collectively, the elevated PaCO_2_ and FiO_2_ requirements observed in both studies underscore the clinical relevance of dead space ventilation and highlight the importance of incorporating physiologic indicators such as VD/VT when evaluating weaning readiness, particularly in hypercapnic patients.

Our findings align with those of Chuang et al., who reported a mean VD/VT ratio of approximately 0.43 in COPD patients, emphasizing its role in identifying impaired gas exchange during physiological stress [29]. In our study, both Type 1 and Type 2 respiratory failure groups had mean VD/VT values below 0.50, which supports the use of this index as a physiologic marker of ventilatory efficiency in the weaning process. The lower VD/VT observed in our Type 1 patients may reflect better ventilation–perfusion matching compared to hypercapnic individuals.

In contrast to the findings of Ghiasi et al., who reported RSBI as a reliable predictor of weaning success while VD/VT had no significant prognostic value, our study found no significant difference in RSBI between respiratory failure types [30]. However, VD/VT was significantly higher in patients with Type 2 respiratory failure, suggesting it may be more informative in hypercapnic patients. These differences may stem from our phenotype-based comparison and emergency department setting, highlighting the need to individualize weaning assessments rather than relying on a single predictor across varied clinical contexts.

Our findings are consistent with the study conducted by Huo et al., which reported that IWI values above 45.7 predicted extubation success with the highest accuracy (AUC: 0.91) and proposed this value as a cutoff [31]. In our study, the mean IWI values in both respiratory failure groups were clearly above this threshold: 79.3 ± 32.5 in the Type 1 group and 70.8 ± 30.7 in the Type 2 group. These high values suggest that patients who underwent successful extubation in our cohort had generally favorable respiratory mechanics, oxygenation, and inspiratory strength. Furthermore, the fact that both groups exceeded the proposed cutoff supports the broad applicability of IWI as a predictive index for weaning success across different respiratory failure phenotypes.

The role of RSBI in predicting extubation success has shown variability in previous studies. For instance, Turhan et al. evaluated serial RSBI measurements and found that patients with later RSBI values ≤72 had approximately a tenfold increase in extubation success [32]. Despite all patients in our study being successfully weaned, the PaO_2_/FiO_2_ ratio in the Type 2 respiratory failure group (169 ± 49.6) closely resembled the values reported for extubation failure in Kwack et al.’s study (174.3 ± 75.6) and was substantially lower than their successful group (231.6 ± 98.1) [33]. Similarly, Bhalla et al. found that lower PaO_2_/FiO_2_ ratios were independently associated with extubation failure, further underscoring its prognostic importance in ventilatory decision-making [34]. This finding suggests that conventional gas exchange markers alone may not adequately capture extubation readiness in hypercapnic patients. In contrast, the VD/VT ratio—an indicator of ventilatory efficiency—was significantly higher in Type 2 patients (0.37 ± 0.04), supporting its role as a more sensitive physiologic marker of impaired gas exchange. These results highlight the importance of integrating VD/VT alongside traditional parameters to improve weaning assessment, particularly in patient populations prone to ventilation-perfusion mismatch and CO_2_ retention.

### Strengths and Limitations

This study has several limitations that should be acknowledged. First, the relatively small sample size—particularly in the Type 2 respiratory failure group—may limit the generalizability of the findings. Additionally, since the study population consisted exclusively of successfully extubated patients, our aim was not to assess the predictive performance of the weaning indices, but rather to examine whether these indices differ between respiratory failure phenotypes. Second, the study was conducted in a single center, which may reduce external validity across diverse clinical settings. Third, while the study focused on immediate weaning outcomes, it did not include post-extubation follow-up data or assess long-term respiratory support requirements. Fourth, the predominance of male patients is likely due to the characteristics of the local population, as the city is known for its ceramics industry. Pulmonary diseases such as pneumonia, COPD, and interstitial lung disease are more frequently observed among factory workers. Fifth, respiratory parameters such as Maximum Inspiratory Pressure (MIP) could not be measured. Moreover, the inclusion of only successfully weaned patients represents a significant limitation, as it precludes any assessment of the diagnostic accuracy of the evaluated indices—including sensitivity, specificity, and optimal cut-off values—which are essential for their clinical applicability and generalizability. Despite these limitations, the findings provide a preliminary foundation for future large-scale, phenotype-specific research in ventilator weaning strategies.

## 5. Conclusions

This study demonstrated that although RSBI and IWI values were similar between Type 1 and Type 2 respiratory failure patients, those with Type 2 RF exhibited significantly higher VD/VT ratios and lower PaO_2_/FiO_2_ levels, indicating impaired gas exchange and reduced alveolar ventilatory efficiency.

These findings highlight the added value of incorporating VD/VT into the clinical assessment of weaning readiness, particularly in hypercapnic patients. Integrating such physiologic markers alongside traditional indices may improve weaning precision and outcomes. Future research with larger cohorts is warranted to validate these results and refine weaning strategies based on respiratory failure phenotypes.

## Figures and Tables

**Table 1:** Demographic Characteristics and Vital Signs of Patients.

		Type 1 RF (n:23)	Type 2 RF (n:12)	*p*-Value
Age (mean ± SD)		72.57 ± 18.2	71.75 ± 8.7	0.88 *
Gender	Male (%)	16 (69.6)	10 (83.3)	0.37 **
	Female (%)	7 (30.4)	2 (16.7)	
Heart Rate (bpm/min)		97.52 ± 14.19	88.25 ± 14.34	0.07 *
SBP (mmHg)		122.96 ± 14.59	130.17 ± 21.95	0.25 *
RR (breath/min)		17.39 ± 2.85	17.00 ± 2.89	0.70 *
Hemoglobin (gr/dL)		11.93 ± 3.21	12.90 ± 2.97	0.39 *
BUN (mg/dL)		28.00 (21.5–37.5)	37.00 (20.3–43.8)	0.70 ***
Creatinine (mg/dL)		1.16 (0.81–1.47)	0.97 (0.85–1.13)	0.56 ***

* Student *t* test (mean ± standard deviation), ** chi-square test (%), *** Mann–Whitney U (median, IQR 25–75) IQR: Inter Quantile Range, RF: Respiratory Failure, BUN: Blood Urea Nitrogen, SBP: Systolic Blood Pressure, RR: Respiratory rate.

**Table 2:** Comparison of mechanical ventilation parameters and arterial blood gas results.

	Type 1 RF (n:23)	Type 2 RF (n:12)	*p*-Value
PEEP (cmH_2_O)	5.0 (5.0–5.5)	5.00 (5.00–5.50)	0.82 **
Pmax (cmH_2_O)	24.0(23.0–25.5)	26.50 (24.50–29.30)	0.16 **
Pplato (cmH_2_O)	18.9 (17.0–20.5)	21.50 (17.00–23.00)	0.14 **
FiO_2_ (%)	30.0(25.5–35.0)	37.50 (25.50–40.00)	0.03 **
V_T_ (mL)	423(400–450)	421 (425–450)	0.76 **
Cdyn (mL/cmH_2_O)	22.0 ± 3.85	20.8 ± 3.87	0.39 *
Cstat (mL/cmH_2_O)	32.5 ± 5.93	30.0 ± 8.17	0.31 *
Ph	7.36 ± 0.04	7.38 ± 0.05	0.37 *
PaO_2_ (mm/Hg)	70.1 ± 13.6	61.1 ± 14.2	0.07 *
PaCO_2_ (mm/Hg)	40.3 ± 4.49	49.1 ± 9.65	<0.001 *
V_E_ (L/min)	7.03 ± 1.02	7.21 ± 1.19	0.65 *
EtCO_2_ (mm/Hg)	28.5 ± 6.51	30.6 ± 6.57	0.38 *

* Student *t* test (mean ± standard deviation), ** Mann–Whitney U ((median, (IQR 25–75)), IQR: Inter Quantile Range, PEEP: Positive End-Expiratory Pressure, Pmax: Maximum Pressure, Pplato: Plateau Pressure, FiO_2_: Fraction of Inspired Oxygen, V_T_: Tidal Volume, Cdyn: Dynamic Compliance, Cstat: Static Compliance, PaO_2_:Partial arterial oxygen pressure PaCO_2_:Partial arterial carbon dioxide pressure, V_E_: Minute Ventilation, EtCO_2_: End-Tidal CO_2_.

**Table 3:** Comparison of Weaning Index Scores.

Weaning Indexes	Type 1 RF (n:23)	Type 2 RF (n:12)	*p*-Value
RSBI (bpm/L)	40.0 (35.0–40.0)	40.0 (35.0–40.0)	1.00 **
V_D_/V_T_	0.29 ± 0.13	0.37 ± 0.04	0.046 *
PaO_2_/FiO_2_ (mmHg)	244 ± 95.6	169 ± 49.6	0.017 *
IWI (bpm/mL)	79.3 ± 32.5	70.8 ± 30.7	0.45 *
PaO_2_/PAO_2_	0.45 (0.36–0.69)	0.33 (0.29–0.39)	0.053 *
GCS	15 (14–15)	15 (14–15)	0.78 **

* Student *t* test (mean ± standard deviation), ** Mann–Whitney U (median, (IQR 25–75)), IQR: Inter Quantile Range, RF: Respiratory Failure, RSBI: Rapid Shallow Breathing Index, V_D_/V_T_: Dead Space to Tidal Volume Ratio, IWI: Integrative Weaning Index, PaO_2_: Partial arterial oxygen pressure, PAO_2_: Partial arterial alveolar pressure, GCS: Glasgow Coma Scale.

## Data Availability

The datasets used and/or analyzed during the current study are available from the corresponding author on reasonable request.

## Abbreviations

The following abbreviations are used in this manuscript:

ABG	Arterial Blood Gas
ARDS	Acute Respiratory Distress Syndrome
BUN	Blood Urea Nitrogen
CDyn	Dynamic Compliance
CStat	Static Compliance
COPD	Chronic Obstructive Pulmonary Disease
ED	Emergency Department
EtCO_2_	End-Tidal Carbon Dioxide
VD/VT	Dead Space to Tidal Volume Ratio
FiO_2_	Fraction of Inspired Oxygen
Hb	Hemoglobin
IWI	Integrative Weaning Index
MV	Mechanical Ventilation
OHS	Obesity Hypoventilation Syndrome
PaCO_2_	Partial Arterial Carbon Dioxide Pressure
PaO_2_	Partial Arterial Oxygen Pressure
PAO_2_	Partial Alveolar Oxygen Pressure
PEEP	Positive End-Expiratory Pressure
Pmax	Peak Inspiratory Pressure
Pplato	Plateau Pressure
RF	Respiratory Failure
RR	Respiratory Rate
RSBI	Rapid Shallow Breathing Index
